# Supplementary material for: Phylogenetic Analyses of Some Key Genes Provide Information on Pollinator Attraction in Solanaceae
Source: Genes (Basel). 2022 Dec 3;13(12):2278. doi: 10.3390/genes13122278 (PMC9778481; doi:10.3390/genes13122278)
Supplement: Supplementary file 1 [file genes-13-02278-s001.zip › genes-2024609-supplementary.pdf]

Phylogenetic analyses of some key genes provide information on pollinator attraction in Solanaceae

Aléxia Gonçalves Pereira[1], Sebastián Guzmán-Rodriguez[1], Loreta B. Freitas[1,*]

[1]Laboratory of Molecular Evolution, Department of Genetics, Universidade Federal do Rio Grande do Sul, Porto Alegre, RS 91501-970, Brazil.

[*]Corresponding author: Department of Genetics, UFRGS, P.O. Box 15053, Porto Alegre, RS 91501-970, Brazil. Fax: +55 51 3308 9823. E-mail: loreta.freitas@ufrgs.br (L.B. Freitas).

Supplementary material

Table S1 Accession numbers for the CDS sequences of the DFR, ODO1, MYB-FL, and NEC 1 genes, used in this study. The colors indicate de query sequence used in the BLAST. The species color matches the sequence code in the database

Species	Gene	*Query*	CDS Access number	Gene Size (pb)	CDS Size (pb)	Database
Petunia axillaris	DFR	X79723.1	Peaxi162Scf00366g00630.1	1779	1143	Sol Genomics
Petunia exserta	DFR	X79723.1	lcl|ORF15/ Peex113Ctg18167g00015.1	1699	1143	Transcriptome
Petunia hybrida (query)	DFR	X79723.1	X79723.1	3879	1143	NCBI
Petunia inflata	DFR	X79723.1	Peinf101Scf00073g04027.1	1134	1143	Sol Genomics
Petunia secreta	DFR	X79723.1	lcl|ORF6 e Psec_k119Scf2421385g0000020.1	-	1143	Transcriptome
Capsicum annuum	DFR	X79723.1	CA02g22270	1584	1047	Sol Genomics
Capsicum annuum var. glabriusculum	DFR	X79723.1	Capang08g000739	-	1146	Sol Genomics
Capsicum annuum var. zunla	DFR	X79723.1	Capana02g002763	-	1149	Sol Genomics
Nicotiana attenuata	DFR	X79723.1	NIATv7_g64584.t1	1418	1143	Sol Genomics
Nicotiana benthamiana 1	DFR	X79723.1	Niben101Scf00606g02015.1	1201	1201	Sol Genomics
Nicotiana benthamiana 2	DFR	X79723.1	Niben101Scf00305g05035.1	1186	1186	Sol Genomics
Nicotiana sylvestris	DFR	X79723.1	mRNA_57456_cds	1467	1146	Sol Genomics
Nicotiana tabacum 1 (TN90)	DFR	X79723.1	mRNA_13452	1492	1478	Sol Genomics
Nicotiana tabacum 2 (TN90)	DFR	X79723.1	mRNA_43521	1488	1146	Sol Genomics
Nicotiana tomentosiformis	DFR	X79723.1	mRNA_5184_cds	1815	1149	Sol Genomics
Sollanum lycopersicum	DFR	X79723.1	Solyc02g085020.4.1	1844	1140	Sol Genomics
Solanum melogenta	DFR	X79723.1	Sme2.5_01401.1_g00004.1	-	1149	Sol Genomics

Solanum pennellii	DFR	X79723.1	Sopen02g029720	2042	1149	Sol Genomics
Solanum pimoinellifolium	DFR	X79723.1	Sopim02g085020.0.1	-	1140	Sol Genomics
Solanum tuberosum (PGSC)	DFR	X79723.1	PGSC0003DMC400006441	-	1149	Sol Genomics
Arabdopsis thaliana	DFR	X79723.1	AT5G42800.1 / NM_123645	1520	1149	TAIR
Petunia axillaris	ODO1	AY705977	Pax_162mrnav4_ODO1_	1520	885	Sol Genomics
Petunia exserta	ODO1	AY705977	Mich_Pex_ODO1	1374	885	Transcriptome
Petunia x hybrida (query)	ODO1	AY705977	AY705977	1535	885	NCBI
Petunia inflata	ODO1	AY705977	Peinf101Scf00284g00012.1	1700	618	Sol Genomics
Petunia secreta	ODO1	AY705977	Psec_k119_mak_ODO1	1397	885	Transcriptome
Capsicum annuum	ODO1	AY705977	CA01g04550	2163	837	Sol Genomics
Capsicum annuum var. glabriusculum	ODO1	AY705977	Capang01g000437	-	834	Sol Genomics
Capsicum annuum var. zunla	ODO1	AY705977	Capana01g000495	-	837	Sol Genomics
Nicotiana attenuata	ODO1	AY705977	NIATv7_g05044.t1	1460	855	Sol Genomics
Nicotiana benthamiana 1	ODO1	AY705977	Niben101Scf15721g00003.1	1617	855	Sol Genomics
Nicotiana benthamiana 2	ODO1	AY705977	Niben101Scf01732g02016.1	1612	855	Sol Genomics
Nicotiana sylvestis	ODO1	AY705977	mRNA_27607_cds	1492	855	Sol Genomics
Nicotiana tabacum 1 (TN90)	ODO1	AY705977	mRNA_184876	1473	846	Sol Genomics
Nicotiana tabacum 2 (TN90)	ODO1	AY705977	mRNA_45796	1472	855	Sol Genomics
Nicotiana tomentosiformis	ODO1	AY705977	mRNA_55682	1719	846	Sol Genomics
Solanum lycopersicum	ODO1	AY705977	Solyc08g079270.3.1	1204	864	Sol Genomics
Solanum melongena	ODO1	AY705977	SMEL_008g317320.1.01	2322	861	Sol Genomics
Solanum penneellii	ODO1	AY705977	Sopen08g027720	-	867	Sol Genomics

Solamum pimpinellifolium	ODO1	AY705977	Sopim08g079270.0.1	-	864	Sol Genomics
Solanum tuberosum (PGSC)	ODO1	AY705977	PGSC0003DMP400008480	-	846	Sol Genomics
Arabdopsis thaliana 1	ODO1	AY705977	AT4G12350.1	1248	861	TAIR
Arabdopsis thaliana 2	ODO1	AY705977	AT4G22680.1	1400	801	TAIR
Petunia axillaris sub. axillaris	MYB-FL	MH732766	KT962949	4573	1056	NCBI
Petunia exserta	MYB-FL	MH732767	KT962950	5502	606	NCBI
Petunia inflata	MYB-FL	MH732766	KT962951	4757	1053	NCBI
Petunia secreta (query)	MYB-FL	MH732766	MH732766/ AZF99108 (prot)	4555	1053	NCBI
Capsicum annuum	MYB-FL	MH732766	CA05g18430	2474	1203	Sol Genomics
Capsicum annuum var. glabriusculum	MYB-FL	CA05g18430	Capang00g002992	-	1020	Sol Genomics
Capsicum annuum var. zunla	MYB-FL	CA05g18430	Capana05g000377	-	1083	Sol Genomics
Nicotiana attenuata 1 (Nicotiana query)	MYB-FL	MH732766	NIATv7_g40261.t1	1171	993	Sol Genomics
Nicotiana attenuata 2	MYB-FL	KT962949	XM_019385552	1217	765	NCBI
Nicotiana benthamiana	MYB-FL	NIATv7_g40261.t1	Niben101Scf08782g00003.1	2658	468	Sol Genomics
Nicotiana sylvestris	MYB-FL	MH732766	XM_009803597	1137	983	NCBI
Nicotiana tabacum 1 (TN90)	MYB-FL	MH732766	mRNA_112793	1238	984	Sol Genomics
Nicotiana tabacum 2 (TN90)	MYB-FL	MH732766	mRNA_164759	1085	888	Sol Genomics
Nicotiana tomentosiformis 1	MYB-FL	MH732766	XM_018774884.1	1065	726	NCBI

Nicotiana tomentosiformis 2	MYB-FL	NIATv7_g40261.t1	mRNA_37095_cds	1126	888	Sol Genomics
Solanum lycopersicum (Solanum query)	MYB-FL	AT5G49330.1	Solyc01g079620.2.1	1143	1017	Sol Genomics
Solanum pennellii	MYB-FL	Solyc01g079620.2.1	Sopen01g031400	2002	1083	Sol Genomics
Solanum pimpinellifolium	MYB-FL	Solyc01g079620.2.1	Sopim01g079620.0.1	-	1017	Sol Genomics
Solanum tuberosum (PGSC)	MYB-FL	Solyc01g079620.2.1	PGSC0003DMP400015892	-	1092	Sol Genomics
Arabdopisis thaliana	MYB-FL	MH732766	AT5G49330.1	1632	1029	TAIR
Petunia axillaris	NEC1	AF313914.1	Peaxi162Scf00303g00004.1	1105	843	Sol Genomics
Petunia exserta	NEC1	AF313914.1	Mich_Pex_NEC1_GBRT01053536.1	1215	797	Transcriptome
Petunia hybrida	NEC1	AF313914.1	AF313914.1/Q9FPN0.1	1182	798	NCBI
Petunia inflata	NEC1	AF313914.1	Peinf101Scf00179g11021.1	1093	843	Sol Genomics
Petunia secreta	NEC1	AF313914.1	Psec_k119_mak_NEC1	477	477	Transcriptome
Capsicum annuum var. zunla	NEC1	AF313914.1	Capana02g000800	-	684	Sol Genomics
Nicotiana attenuata	NEC1	AT2G39060.1	KC832836.1	1296	819	NCBI
Nicotiana benthamiana 1	NEC1	Q9FPN0.1	Niben101Scf02594g03008.1	1010	706	Sol Genomics
Nicotiana benthamiana 2	NEC1	Q9FPN0.1	Niben101Scf04629g03012.1	1057	819	Sol Genomics
Nicotiana sylvestris	NEC1	Q9FPN0.1	mRNA_19339_cds	971	781	Sol Genomics
Nicotiana tabacum 1 (TN90)	NEC1	Q9FPN0.1	mRNA_31109	1000	795	Sol Genomics
Nicotiana tabacum 2 (TN90)	NEC1	Q9FPN0.1	mRNA_68247	1174	795	Sol Genomics
Nicotiana tomentosiformis	NEC1	Q9FPN0.1	mRNA_60304_cds	1235	795	Sol Genomics

Solanum lycopersicum (Solanum query)	NEC1	Q9FPN0.1	Solyc09g074530.3.1	1136	861	Sol Genomics
Solanum melogenta	NEC1	Solyc09g074530.3.1	Sme2.5_06014.1_g00003.1	1613	867	Sol Genomics
Solanum pennellii	NEC1	Solyc09g074530.3.1	Sopen09g029370	-	861	Sol Genomics
Solanum pimpinellifolium	NEC1	Solyc09g074530.3.1	Sopim09g074530.0.1	-	861	Sol Genomics
Solanum tuberosum (PGSC)	NEC1	Solyc09g074530.3.1	PGSC0003DMC400020098	-	864	Sol Genomics
Arabdopsis thaliana	NEC1	AT2G39060.1	AT2G39060.1	902	777	TAIR

Table S2 Accession numbers for the protein sequences of the DFR, ODO1, MYB-FL and NEC 1 genes, used in this study. The colors indicate de query sequence used in the BLAST. The species color matches the sequence code in the database

Species	Gene	*Query*	Protein Access number	Size (aa)	Domain/ HMMER	Database
Petunia axillaris	DFR	P14720	Peaxi162Scf00366g00630.1	380	Epimerase	SolGenomics
Petunia exserta	DFR	P14720	lcl_ORF15	380	Epimerase	Transcriptoma
Petunia hybrida (query)	DFR	P14721	P14720	380	Epimerase	NCBI
Petunia inflata	DFR	P14720	Peinf101Scf00073g04027.1	380	Epimerase	SolGenomics
Petunia secreta	DFR	P14720	lcl_ORF6	380	Epimerase	Transcriptoma
Capsicum annuum	DFR	P14720	CA02g22270	348	Epimerase	SolGenomics
Capsicum annuum var. glabriusculum	DFR	P14720	Capang08g000739	382	Epimerase	SolGenomics
Capsicum annuum var. zunla	DFR	P14720	Capana02g002763	382	Epimerase	SolGenomics
Nicotiana attenuata	DFR	P14720	NIATv7_g64584.t1	380	Epimerase	SolGenomics
Nicotiana benthamiana 1	DFR	NIATv7_g64584.t1	Niben101Scf00606g02015.1	380	Epimerase	SolGenomics
Nicotiana benthamiana 2	DFR	NIATv7_g64584.t1	Niben101Scf00305g05035.1	382	Epimerase	SolGenomics
Nicotiana silvestris	DFR	P14720	mRNA_57456_cds	381	Epimerase	SolGenomics
Nicotiana tabacum 1 (TN90)	DFR	P14722	mRNA_13452	382	Epimerase	SolGenomics
Nicotiana tabacum 2 (TN90)	DFR	P14721	mRNA_43521	381	Epimerase	SolGenomics
Nicotiana tomentosiformis	DFR	P14720	mRNA_5184_cds	382	Epimerase	SolGenomics
Solanum lycopersicum	DFR	P14723	Solyc02g085020.4.1	379	Epimerase	SolGenomics
Solanum melogenta	DFR	P14724	Sme2.5_01401.1_g00004.1	382	Epimerase	SolGenomics
Solanum pennellii	DFR	P14725	Sopen02g029720	382	Epimerase	SolGenomics
Solanum pimoinellifolium	DFR	P14726	Sopim02g085020.0.1	379	Epimerase	SolGenomics
Solanum tuberosum (PGSC)	DFR	P14728	PGSC0003DMC400006441	382	Epimerase	SolGenomics
Arabdopsis thaliana	DFR	P14729	At1g01030	382	Epimerase	TAIR

Petunia axillaris (Gina)	ODO1	AAV98200	Pax_162mrnav4_ODO1_	294	2 Myb-like	Transcriptoma
Petunia exserta (Gina)	ODO1	AAV98200	Mich_Pex_ODO1	294	2 Myb-like	Transcriptoma
Petunia x hybrida	ODO1	AAV98200	AAV98200	294	2 Myb-like	NCBI
Petunia inflata	ODO1	AAV98200	Peinf101Scf00284g00012.1	205	Myb-like	SolGenomics
Petunia secreta (Gina)	ODO1	AAV98200	Psec_k119_mak_ODO1	294	2 Myb-like	SolGenomics
Capsicum annuum	ODO1	AAV98200	CA01g04550	278	2 Myb-like	SolGenomics
Capsicum annuum var. glabriusculum	ODO1	AAV98200	Capang01g000437	278	2 Myb-like	SolGenomics
Capsicum annuum var. zunla	ODO1	AAV98200	Capana01g000495	278	2 Myb-like	SolGenomics
Nicotiana attenuata	ODO1	AAV98200	NIATv7_g05044.t1	284	2 Myb-like	SolGenomics
Nicotiana benthamiana 1	ODO1	AAV98200	Niben101Scf15721g00003.1	284	2 Myb-like	SolGenomics
Nicotiana benthamiana 2	ODO1	AAV98200	Niben101Scf01732g02016.1	284	2 Myb-like	SolGenomics
Nicotiana sylvestis	ODO1	AAV98200	mRNA_27607_cds	284	2 Myb-like	SolGenomics
Nicotiana tabacum 1 (TN90)	ODO1	AAV98200	mRNA_184876	281	2 Myb-like	SolGenomics
Nicotiana tabacum 2 (TN90)	ODO1	AAV98200	mRNA_45796	284	2 Myb-like	SolGenomics
Nicotiana tomentosiformis	ODO1	AAV98200	mRNA_55682	281	2 Myb-like	SolGenomics
Solanum lycopersicum	ODO1	AAV98200	Solyc08g079270.3.1	287	2 Myb-like	SolGenomics
Solanum melongena	ODO1	AAV98200	SMEL_008g317320.1.01	286	2 Myb-like	SolGenomics
Solanum penneellii	ODO1	AAV98200	Sopen08g027720	288	2 Myb-like	SolGenomics
Solamum pimpinellifolium	ODO1	AAV98200	Sopim08g079270.0.1	287	2 Myb-like	SolGenomics
Solanum tuberosum (PGSC)	ODO1	AAV98200	PGSC0003DMP400008480	281	2 Myb-like	SolGenomics
Arabdopsis thaliana 1	ODO1	AAV98200	AT4G12350.1	286	2 Myb-like	TAIR
Arabdopsis thaliana 2	ODO1	AAV98200	AT4G22680.1	266	2 Myb-like	TAIR
Petunia axillaris	MYB-FL	MH732766	ALQ80977	351	2 Myb-like	NCBI
Petunia exserta	MYB-FL	MH732766	ALQ80978.1	201	2 Myb-like	NCBI
Petunia inflata	MYB-FL	MH732766	ALQ80979	351	2 Myb-like	NCBI

Petunia secreta (query)	MYB-FL	MH732766	AZF99108	350	2 Myb-like	NCBI
Capsicum annuum	MYB-FL	MH732766	CA05g18430	400	2 Myb-like	SolGenomics
Capsicum annuum var. glabriusculum	MYB-FL	CA05g18430	Capang00g002992	340	Myb-like	SolGenomics
Capsicum annuum var. zunla	MYB-FL	CA05g18430	Capana05g000377	362	Myb-like	SolGenomics
Nicotiana attenuata 1 (Nicotiana query)	MYB-FL	MH732766	NIATv7_g40261.t1	330	2 Myb-like	SolGenomics
Nicotiana attenuata 2	MYB-FL	KT962949	XP_019241097	329	2 Myb-like	NCBI
Nicotiana benthamiana	MYB-FL	NIATv7_g40261.t1	Niben101Scf08782g00003.1	155	2 Myb-like	SolGenomics
Nicotiana sylvestris	MYB-FL	NIATv7_g40261.t1	mRNA_47970_cds	327	2 Myb-like	NCBI
Nicotiana tabacum 1 (TN90)	MYB-FL	NIATv7_g40261.t1	mRNA_112793	327	2 Myb-like	SolGenomics
Nicotiana tabacum 2 (TN90)	MYB-FL	NIATv7_g40261.t1	mRNA_164759	295	2 Myb-like	SolGenomics
Nicotiana tomentosiformis 1	MYB-FL	MH732766	XP_018630400.1	295	2 Myb-like	NCBI
Nicotiana tomentosiformis 2	MYB-FL	NIATv7_g40261.t1	mRNA_37095_cds	295	2 Myb-like	SolGenomics
Solanum lycopersicum (Solanum query)	MYB-FL	AT5G49330.1	Solyc01g079620.4.1	338	2 Myb-like	SolGenomics
Solanum pennellii	MYB-FL	Solyc01g079620.2.1	Sopen01g031400	360	2 Myb-like	SolGenomics
Solanum pimpinellifolium	MYB-FL	Solyc01g079620.2.1	Sopim01g079620.0.1	338	2 Myb-like	SolGenomics
Solanum tuberosum (PGSC)	MYB-FL	Solyc01g079620.2.1	PGSC0003DMP400015892	363	2 Myb-like	SolGenomics
Arabdopisis thaliana	MYB-FL	MH732766	AT5G49330.1	342	2 Myb-like	TAIR
Petunia axillaris	NEC1	AF313914.1	Peaxi162Scf00303g00004.1	280	2 MtN3_slv	SolGenomics
Petunia exserta	NEC1	AF313914.1	Mich_Pex_NEC1_GBRT01053536.1	265	2 MtN3_slv	Transcriptoma
Petunia hybrida	NEC1	AF313914.1	Q9FPN0.1	265	2 MtN3_slv	NCBI
Petunia inflata	NEC1	Q9FPN0.1	Peinf101Scf00179g11021.1	280	2 MtN3_slv	SolGenomics
Petunia secreta	NEC1	AF313914.1	Psec_k119_mak_NEC1	158	MtN3_slv	Transcriptoma
Capsicum annuum var. zunla	NEC1	Q9FPN0.1	Capana02g000800	227	MtN3_slv	SolGenomics

Nicotiana attenuata	NEC1	AT2G39060.1	AGL98401	272	2	MtN3_slv	NCBI
Nicotiana benthamiana 1	NEC1	AGL98401	Niben101Scf02594g03008.1	236	2	MtN3_slv	SolGenomics
Nicotiana benthamiana 2	NEC1	AGL98401	Niben101Scf04629g03012.1	272	2	MtN3_slv	SolGenomics
Nicotiana sylvestris	NEC1	AGL98401	mRNA_19339 gene_11121	263	2	MtN3_slv	SolGenomiscs
Nicotiana tabacum 1 (TN90)	NEC1	AT2G39060.1	mRNA_31109	263	2	MtN3_slv	SolGenomics
Nicotiana tabacum 2 (TN90)	NEC1	AT2G39060.1	mRNA_68247	264	2	MtN3_slv	SolGenomics
Nicotiana tomentosiformis	NEC1	AGL98401	mRNA_60304_cds	264	2	MtN3_slv	SolGenomiscs
Solanum lycopersicum (Solanum query)	NEC1	Q9FPN0.1	Solyc09g074530.3.1	286	2	MtN3_slv	SolGenomics
Solanum melogenta	NEC1	Solyc09g074530.3.1	Sme2.5_06014.1_g00003.1	288	2	MtN3_slv	SolGenomics
Solanum pennellii	NEC1	Solyc09g074530.3.1	Sopen09g029370	286	2	MtN3_slv	SolGenomics
Solanum pimpinellifolium	NEC1	Solyc09g074530.3.1	Sopim09g074530.0.1	286	2	MtN3_slv	SolGenomics
Solanum tuberosum (PGSC)	NEC1	Solyc09g074530.3.1	PGSC0003DMC400020098	287	2	MtN3_slv	SolGenomics
Arabdopisis thaliana	NEC1	AF313914.1	AT2G39060.1	258	2	MtN3_slv	TAIR

Table S3 Parameter estimates for Site Model and log-likelihood values under-tested models. Values highlighted in bold indicate relevant values for the interpretation of the result. "np" stands for the number of parameters. Ln (Likelihood) refers to the log value of the likelihood

Model	np	Ln L	Estimates of parameters				Model compared	LRT P-value	Positive sites
DFR									
			p:	0.803	0.197	0.000			
M3	46	-4088.738162	ω:	0.055	0.745	28.480			
M0	42	-4155.312225	ω0:	0.151			M0 vs. M3	**0.000**	Not Allowed
			p:	0.847	0.083	0.069			
M2a	45	-4089.491462	ω:	0.066	1.000	1.000			
			**p:**	**0.847**	0.153				
M1a	43	-4089.491456	**ω:**	**0.066**	1.000		M1a vs. M2a	**1.000**	Not Allowed
			p0:	0.922					
			p:	0.447					
			p1	0.078					
			q:	3.366					
M8	45	-4080.601886	ω:	1.344					
M7	43	-4089.772926	p:	0.288					
			q:	1.069			M7 vs.M8	0.000	Not Allowed
MYB-FL									
			p:	0.861	0.074	0.065			
M3	44	-868.644773	ω:	0.058	0.415	0.920			
M0	40	-882.433594	ω0:	0.107			M0 vs. M3	**0.000**	Not Allowed
M2a	43	-869.048348	p:	0.903	0.031	0.066	M1a vs. M2a	**1.000**	Not Allowed

			ω:	0.069	1.000	1.000			
			**p:**	**0.903**	0.097				
M1a	41	-869.048318	**ω:**	**0.069**	1.000				
			p0:	0.918					
			p:	1.725					
			p1	0.081					
			q:	20.192					
M8	43	-868.869537	ω:	1.000					
			p:	0.460					
M7	41	-972.778403	q:	2.517			M7 vs.M8	0.020	Not Allowed
ODO1									
			p:	0.547	0.408	0.045			
M3	48	-1870.452557	ω:	0.000	0.168	1.024			
M0	44	-1913.442537	ω0:	0.088			M0 vs. M3	**0.000**	Not Allowed
			p:	0.893	0.078	0.030			
M2a	47	-1885.179329	ω:	0.053	1.000	1.000			
			**p:**	**0.893**	0.107				
M1a	45	-1885.179329	**ω:**	**0.053**	1.000		M1a vs. M2a	**1.000**	Not Allowed
			p0:	0.958					
			p:	0.201					
			p1	0.041					
			q:	1.931					
M8	45	-1871.24	ω:	1.073					
			p:	0.226					
M7	46	-1875.46	q:	1.539			M7 vs.M8	0.014	Not Allowed
NEC1									

			p:	0.337	0.434	0.229			
M3	42	-1899.902013	ω:	0.024	0.209	0.697			
M0	38	-1930.145943	ω0:	0.221			M0 vs. M3	**0.000**	Not Allowed
			p:	0.739	0.185	0.075			
M2a	41	-1906.20185	ω:	0.121	1.000	1.000			
			**p:**	**0.739**	0.261				
M1a	39	-1906.20185	**ω:**	**0.121**	1.000		M1a vs. M2a	**1.000**	Not Allowed
			p0:	0.920					
			p:	0.613					
			p1	0.079					
			q:	2.195					
M8	41	-1900.113914	ω:	1.000					
			p:	0.526					
M7	39	-1900.00223	q:	1.418			M7 vs.M8	0.852	Not Allowed

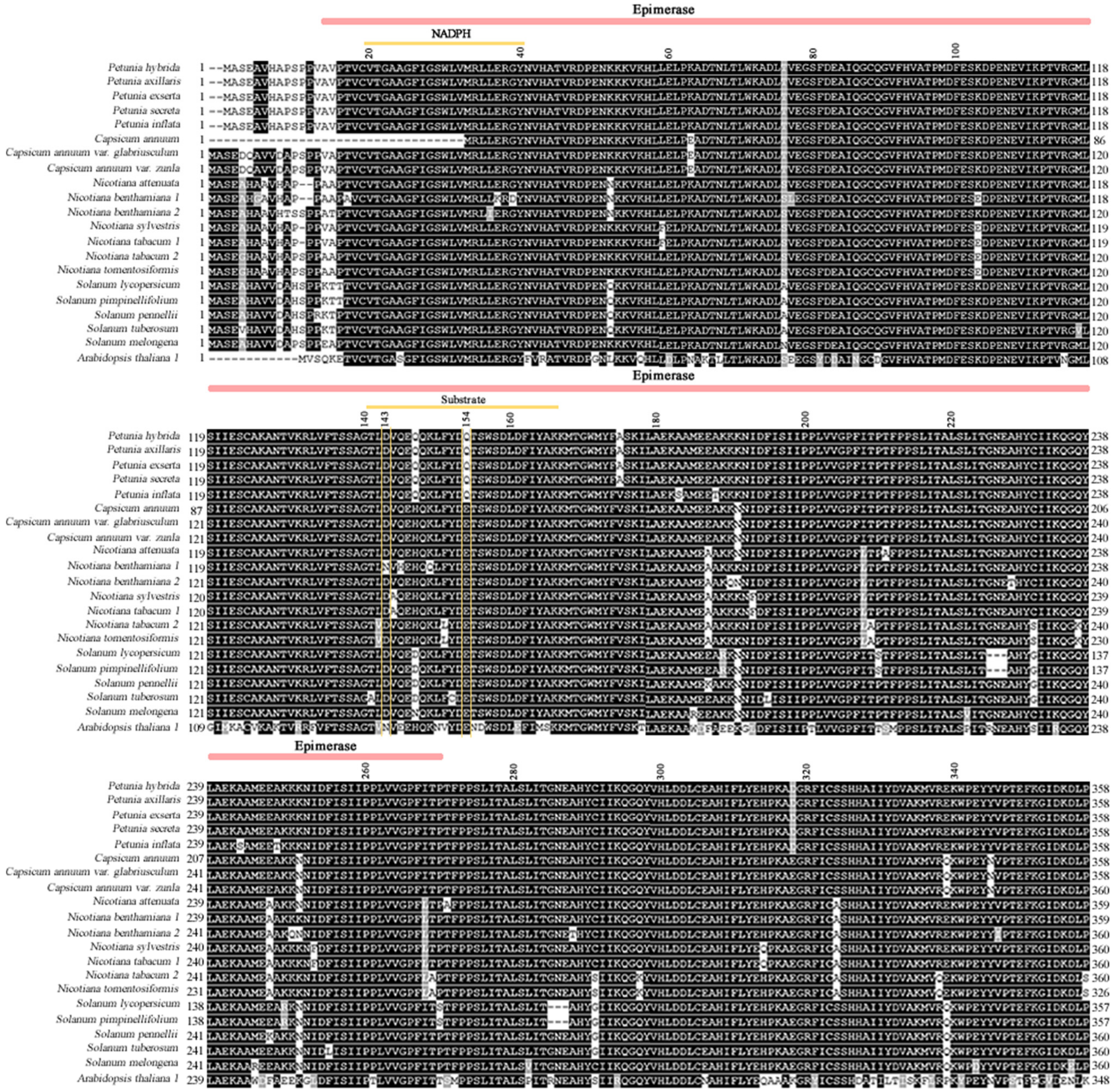

Figure S1. Multiple sequence alignment of DFR protein domain in Solanaceae species. Dark boxes indicate conserved amino acids. Colored lines above the sequences indicated epimerase domain (ligh pink line), NADP(H)-binding site and the substrate specificity site (yellow line). Amino acid position 143 e 154 highlighted in yellow indicate important sites to substrate specificity and enzime activity, respectively.

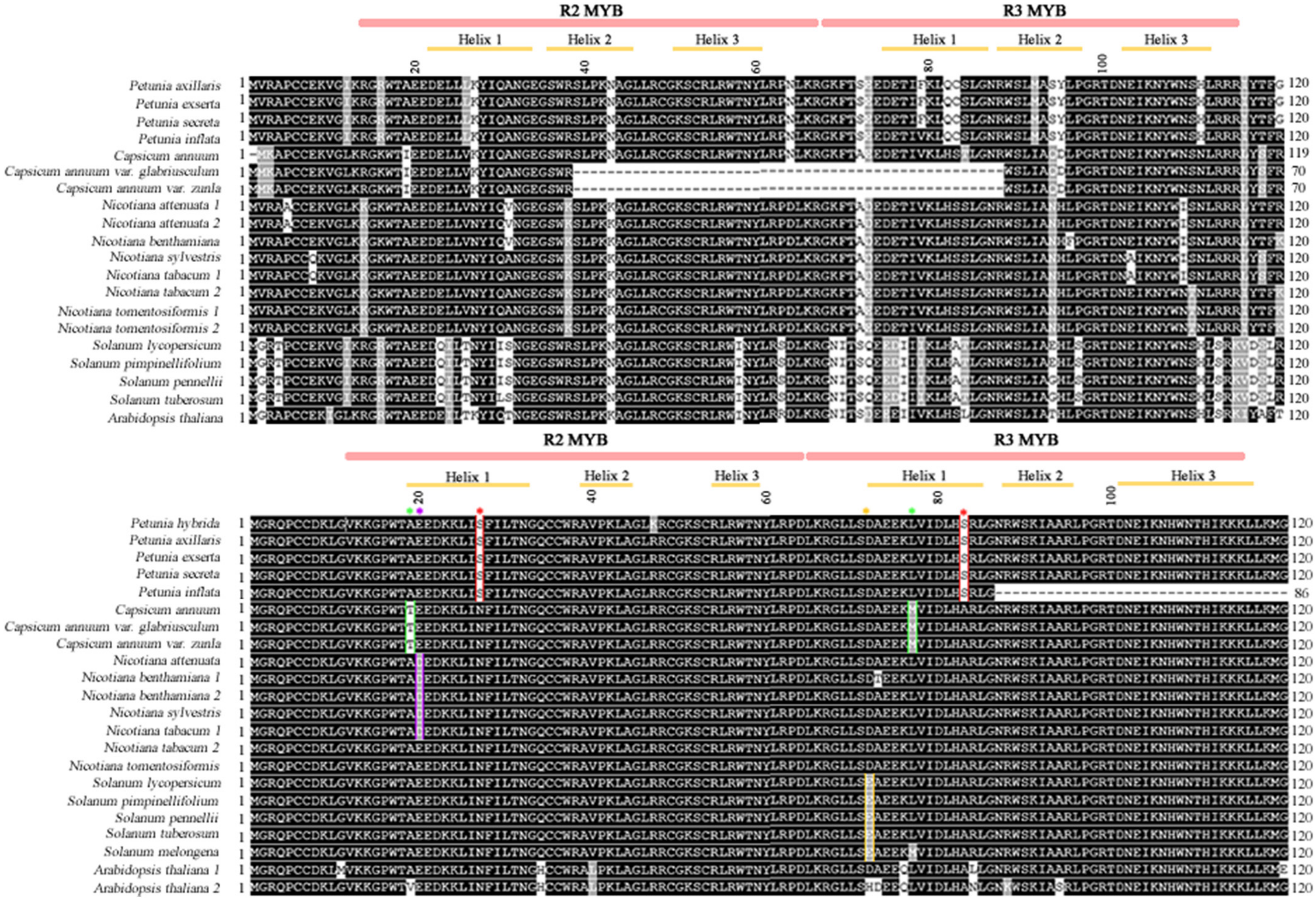

Figure S2. Multiple sequence alignment of MYB-FL (A) and ODO1 (B) R2R3 protein domain in Solanaceae species. Dark boxes indicate conserved amino acids. Colored lines above the sequences indicated the R2R3 domains (ligh pink line) and the helices structure (yellow line). Symbols (*) indicate each genus exclusive substitutions [*Petunia* (red), *Capsicum* (green), *Nicotiana* (purple), and *Solanum* (yellow)]. All substitutions occur within helix 1 of the R2R3 domain.

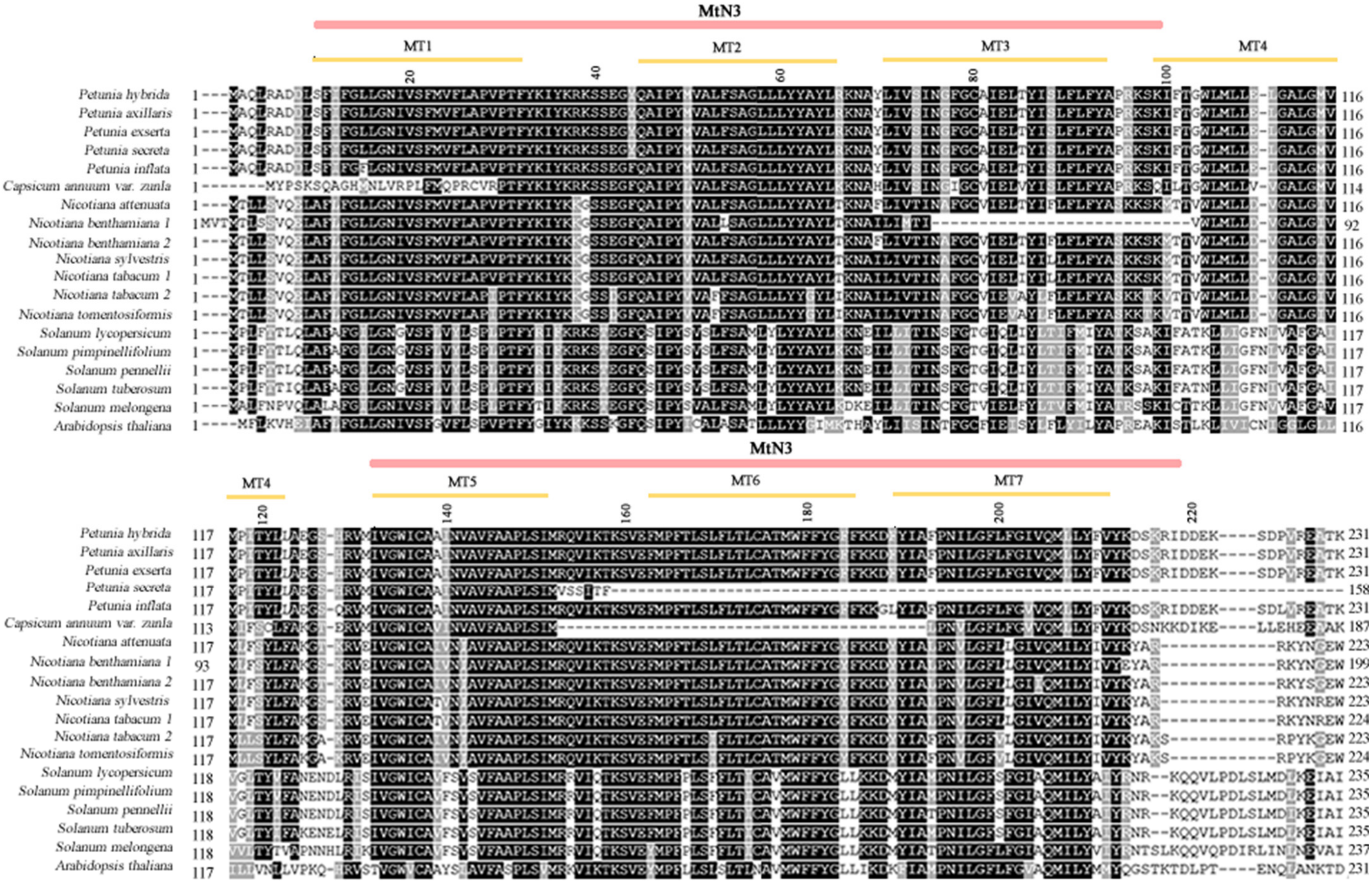

Figure S3. Multiple sequence alignment of the NEC1 MtN3 protein domain in Solanaceae species. Dark boxes indicate conserved amino acids. Colored lines above the sequences indicated the MtN3 domain (ligh pink line) that consist of two tandemly repeated 3-TMs separated by a single TM (yellow line).

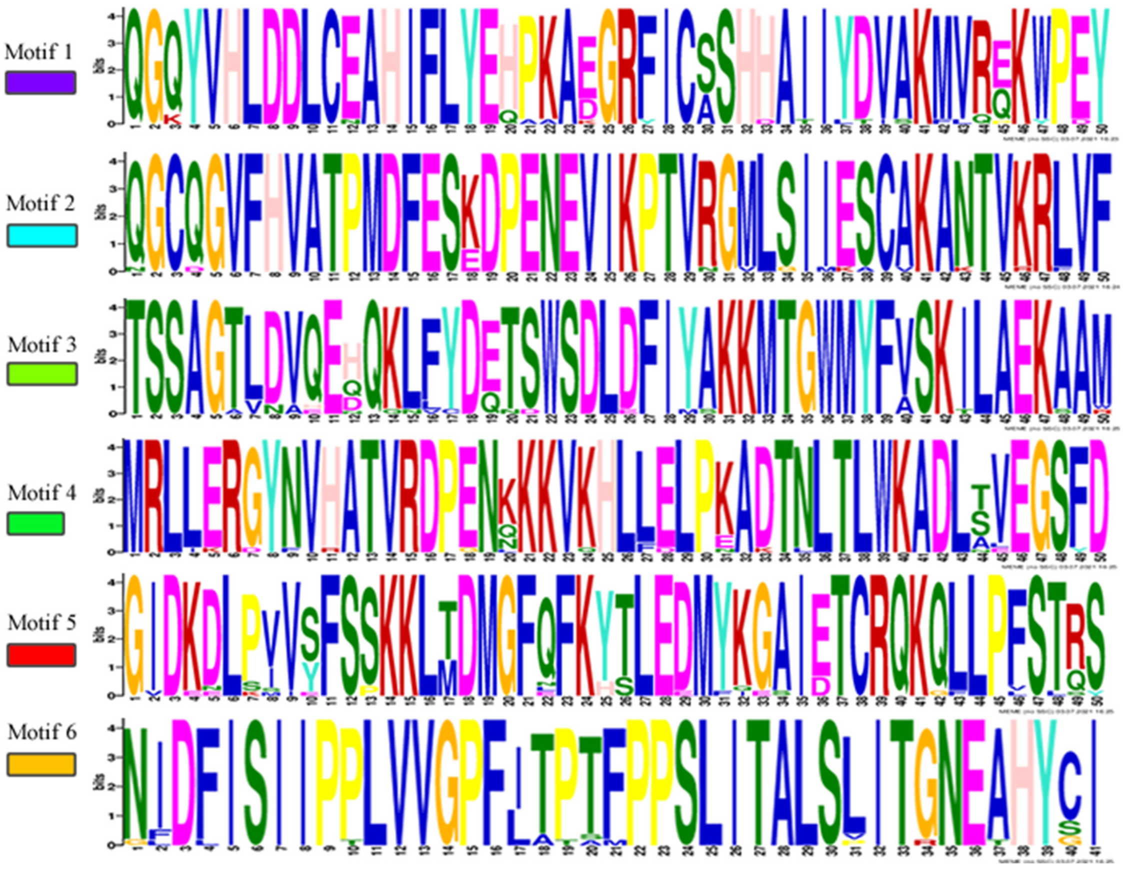

Figure S4. Logo of conserved motifs of DFR protein sequences. Numbered and colored boxes indicate the motifs identified in MEME analyze. Each motif has a corresponding amino acid logo. Large letters indicate the conserved and small letters indicate variable positions, with variations also indicated in the last.

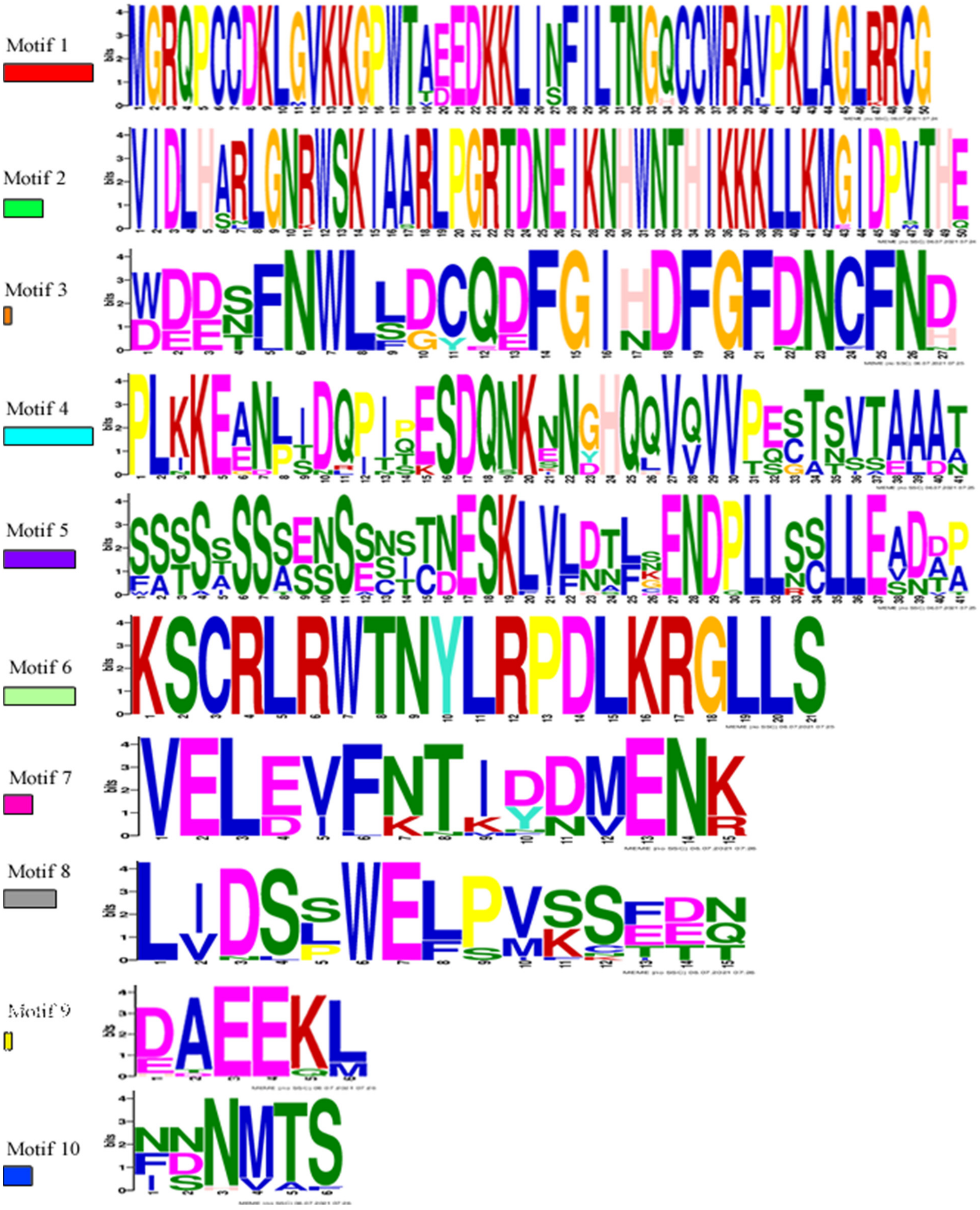

Figure S5. Logo of conserved motifs of ODO1 protein sequences. Numbered and colored boxes indicate the motifs identified in MEME analyze. Each motif has a corresponding amino acid logo. Large letters indicate the conserved and small letters indicate variable positions, with variations also indicated in the last.

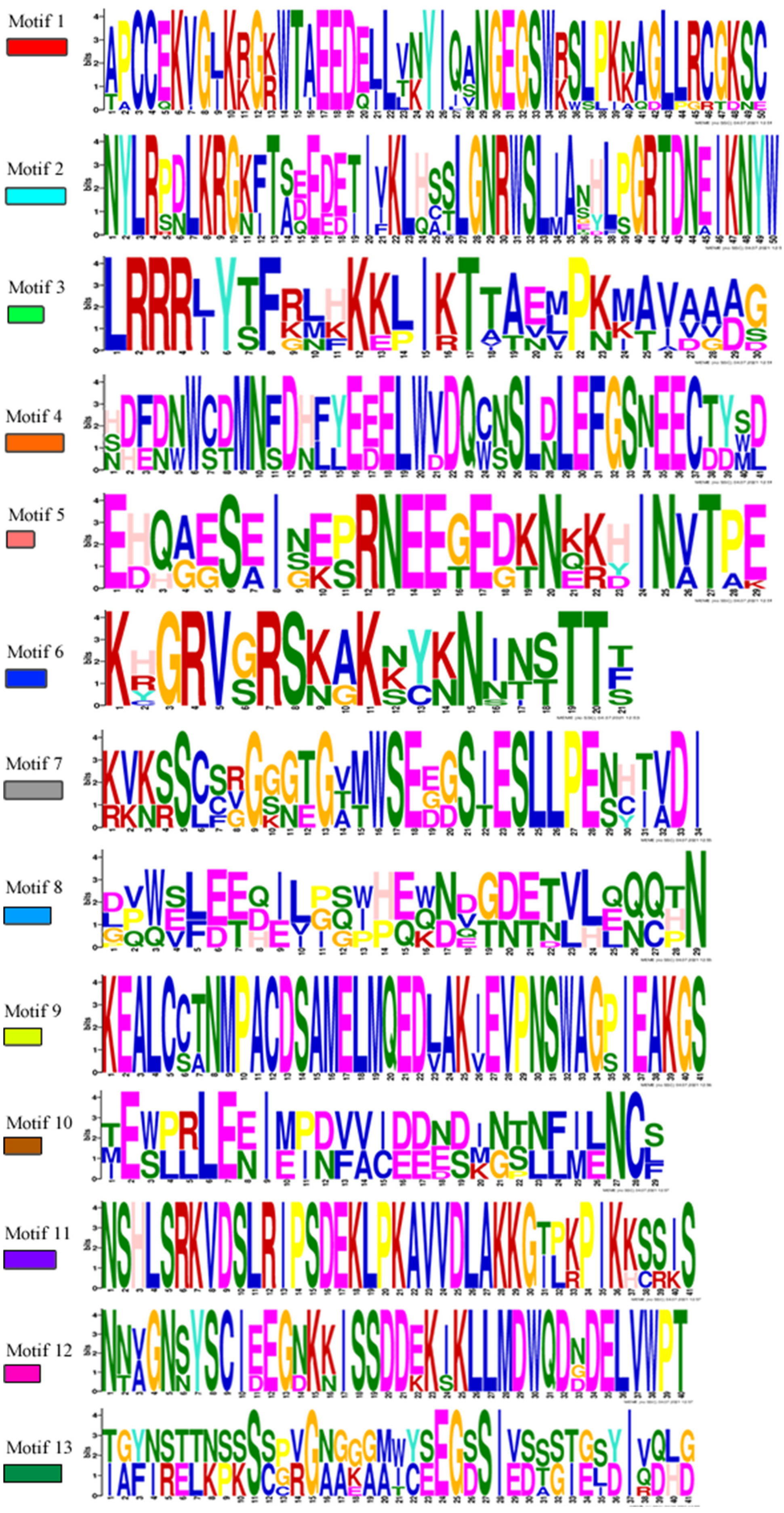
Motif 1
Motif 2
Motif 3
Motif 4
Motif 5
Motif 6
Motif 7
Motif 8
Motif 9
Motif 10
Motif 11
Motif 12
Motif 13

Figure S6. Logo of conserved motifs of MYB-FL protein sequences. Numbered and colored boxes indicate the motifs identified in MEME analyze. Each motif has a corresponding amino acid logo. Large letters indicate the conserved and small letters indicate variable positions, with variations also indicated in the last.

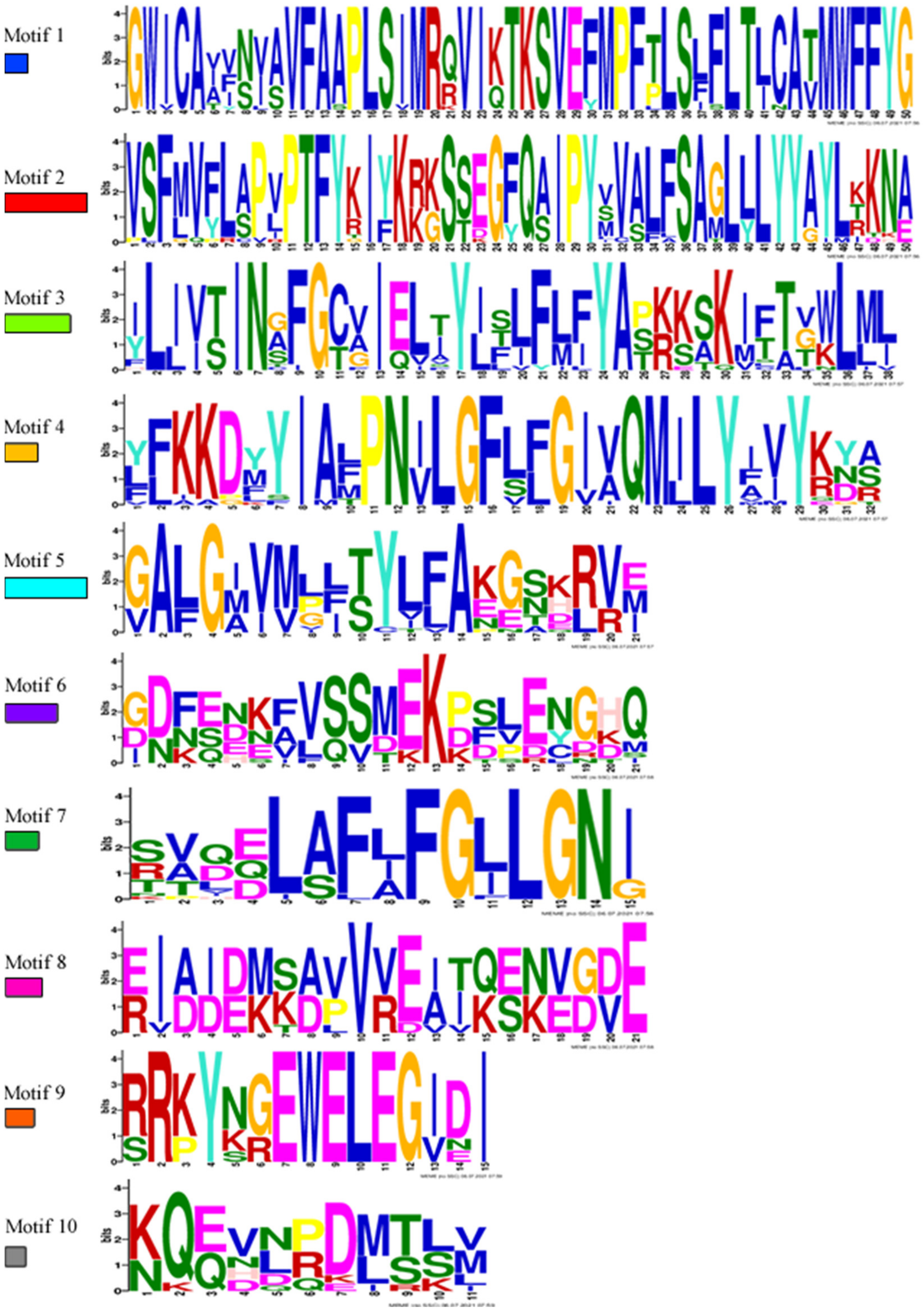

Figure S7. Logo of conserved motifs of NEC1 protein sequences. Numbered and colored boxes indicate the motifs identified in MEME analyze. Each motif has a corresponding amino acid logo. Large letters indicate the conserved and small letters indicate variable positions, with variations also indicated in the last.
